# Impact of rapid lactate clearance as an indicator of hemodynamic optimization on outcome in out-of-hospital cardiac arrest: A retrospective analysis

**DOI:** 10.1371/journal.pone.0214547

**Published:** 2019-04-01

**Authors:** Kap Su Han, Su Jin Kim, Eui Jung Lee, Kyoung Yae Park, Ji Young Lee, Sung Woo Lee

**Affiliations:** Department of Emergency Medicine, College of Medicine, Korea University, Inchon-ro 73, Seongbuk-gu, Seoul, Republic of Korea; Erasmus Medical Center, NETHERLANDS

## Abstract

**Objective:**

We analyzed the impact of rapid lactate clearance as an indicator of hemodynamic optimization on the outcome of out-of-hospital cardiac arrest (OHCA) during early post-cardiac arrest care (PCAC).

**Methods:**

This retrospective analysis was based on a prospective cohort. We selected adult patients with OHCA who achieved a survival event between May 2006 and December 2017. Serum lactate levels were measured at 0 and 6 h after a survival event. The lactate clearance rate was calculated as (lactate at 0 h—lactate at 6 h)/lactate at 0 h. The threshold lactate clearance value for predicting survival to discharge was determined by receiver operating characteristic (ROC) curve analysis. Rapid lactate clearance was defined as the lactate clearance above the threshold value or normalization of serum lactate level (<2.0 mmol/L) at 6 h after a survival event. The primary outcome was survival to discharge. Multivariate logistic regression was used to predict survival to discharge.

**Results:**

We enrolled 335 patients. 145 patients (43.3%) survived at discharge. The threshold value of lactate clearance was ≥34% to predict survival to discharge. After adjustment for confounders, the adjusted odds ratios for no hypotension at both 0 and 6 hours and for the presence of rapid lactate clearance for survival to discharge were 8.018 (95% confidence interval [CI] 2.802–22.947) and 2.193 (95% CI 1.263–3.808), respectively. Among patients with early recovery from initial hypotension or with no hypotension events at both 0 and 6 hours, the survival rates were significantly higher in the rapid lactate clearance group than in the non-rapid lactate clearance group.

**Conclusions:**

During the early PCAC period, a rapid decrease in serum lactate level was an indicator of good outcome. Hemodynamic optimization including not only prevention and immediate correction of hypotension but also rapid lactate clearance should be considered in OHCA patients.

## Introduction

Systemic hypotension and inadequate tissue perfusion are associated with mortality and commonly occur during post-cardiac arrest periods [1]. The level of serum lactate reflects the status of tissue perfusion and is a prognostic marker for global tissue hypoxia in case of circulatory shock or arrest [2]. Though the use of single, isolated levels of lactate for the prediction of outcomes has limitations, serial measurement of serum lactate levels is a suitable predictor of patient outcomes. Early recovery from hyperlactatemia or a high lactate clearance rate during the early post-cardiac arrest care (PCAC) period are considered good predictive factors [3–6]. Hemodynamic optimization within an adequate time period after an arrest is important for favorable outcomes in case of cardiac arrest [7–9]. The 2015 American Heart Association (AHA) guidelines recommend maintaining an optimal blood pressure to allow improvement of organ and brain perfusion [10], which may result in the reduction of serum lactate levels. Therefore, monitoring the rate of lactate clearance is important for optimization of hemodynamics during the early PCAC period in patients with out-of-hospital cardiac arrest (OHCA). We hypothesized that 1) rapid lactate clearance is a predictor of good outcome in OHCA and 2) patient outcome is poor even if hypotension is prevented or immediately corrected, if not accompanied by rapid lactate clearance. Thus, we aimed to assess the impact of rapid lactate clearance as an indicator of hemodynamic optimization on good neurologic outcomes during the early PCAC period in patients with OHCA.

## Materials and methods

### Study design and setting

This retrospective analysis based on a prospective cohort was conducted at the emergency department (ED) of Korea University Anam Hospital from May 2006 to December 2017. We analyzed the cardiopulmonary resuscitation (CPR) registry comprising prospectively collected data on pre- and in-hospital variables of patients with cardiac arrest who received CPR. The Institutional Review Board of Korea University Anam Hospital approved this retrospective analysis separately from the cohort establishment (IRB no. 2018AN0442). The IRB of the Korea University Medical Center waived the requirement for informed consent. The data were analyzed anonymously.

### Collection of data from the CPR registry

A CPR coordinator prospectively collected data from the CPR registry based on Utstein-style guidelines [11]. The registry included demographic data; whether the arrest was witnessed; location of arrest; presumed cause of arrest; presence of comorbidities; incidence of suspected or confirmed trauma; presumed time of arrest; presence of bystander CPR; first documented arrest rhythm by the emergency medical service provider; any return of spontaneous circulation (ROSC); presence of a survival event after advanced cardiac life support (ACLS); presence of extracorporeal CPR (ECPR); application of therapeutic hypothermia (TTM), coronary angiography (CAG), or percutaneous coronary intervention; 24-hour survival; hospital length of stay at discharge; date of death or discharge from the hospital; and cerebral performance category (CPC) score at discharge and at 3 months post-arrest. In addition, this CPR registry included laboratory data and data on vital signs obtained during CPR or within 0 hours to 48 hours after a survival event.

### Management in the post-cardiac arrest period

All patients who experienced cardiac arrest received ACLS from emergency physicians based on the AHA ACLS guidelines. Treatment was performed according to AHA guidelines for the post-cardiac arrest care (PCAC) period. During the PCAC period, we performed mechanical ventilation with a target of 94% oxygen saturation and a carbon dioxide partial pressure of 35–45 mmHg. The hemodynamic goals in the post-cardiac arrest care period are to maintain a mean arterial pressure (MAP) of ≥65 mmHg and systolic blood pressure (SBP) of ≥90 mmHg. The volume status of patients was assessed through echocardiography and physical examination, and blood pressure was maintained using fluid therapy and vasoactive agents such as norepinephrine and dopamine and epinephrine. Before 2015, TTM was conducted at 32–34°C for 24 hours. After the 2015 ACLS guideline change, TTM was conducted at 32–36°C for 24 hours. CAG was performed as soon as possible in cases of suspected acute coronary syndrome (ACS) or unknown origin, and computed tomography (CT) and echocardiography were performed if required.

### Selection of study patients and data analysis

**Fig 1:**
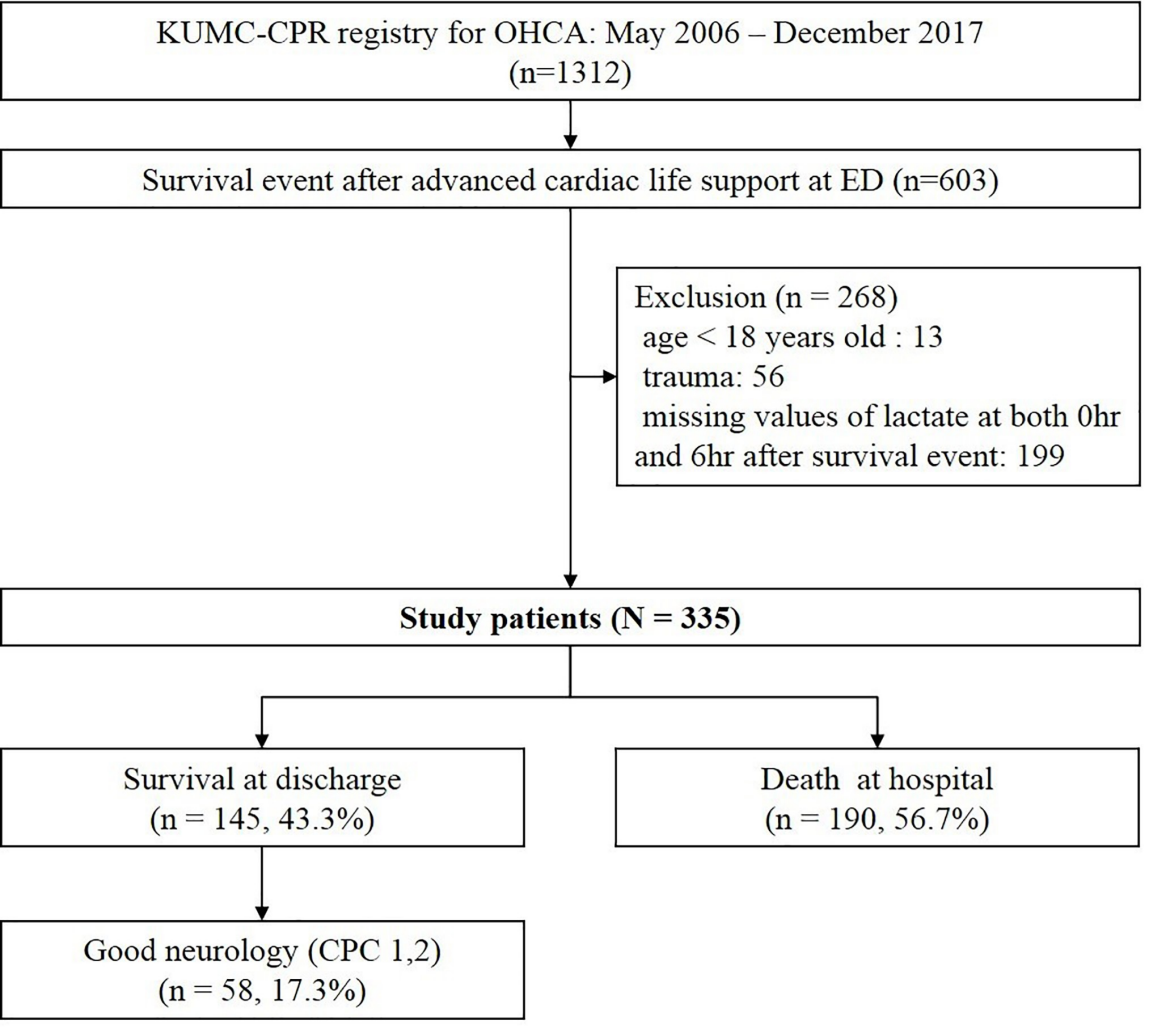
Selection and outcomes of patients included in the study. OHCA, out-of-hospital cardiac arrest; ED, emergency department; CPC, cerebral performance category.

We included patients with OHCA who had a survival event Box 1 and excluded those who received ECPR for refractory arrest. We also excluded patients aged less than 18 years, those who had cardiac arrest after trauma, and those whose lactate values were not recorded at both 0 and 6 hours after a survival event (Fig 1).

**Box 1. Definitions of terminology**

**Table:** 

Survival event	Sustained return of spontaneous circulation (ROSC) ≥ 20 min
ACLS duration	Time interval from the first chest compression provided by healthcare providers to a survival event
Rate of lactate clearance	(Serum lactate level at 0 h—serum lactate level at 6 h)/serum lactate level at 0 h × 100 (%)
Rapid lactate clearance	Rate of lactate clearance above the threshold value (≥34%) or serum lactate level of <2 mmol/L at 6 h after a survival event

The primary outcome was survival to discharge. CPR-related factors between the survivor and non-survivor groups were compared to determine the predictive indicators of good survival outcome. Data on MAP and serum lactate level were gathered at 0 and 6 hours after a survival event. Hypotension was defined as a MAP of <65 mmHg. The lactate clearance rate was calculated as the difference between the lactate level (mmol/L) at 0 and 6 hours after a survival event divided by the lactate level at 0 hours Box 1. We used receiver operator characteristic (ROC) curve analysis to identify the threshold value for the rate of lactate clearance in terms of predicting survival to discharge. To estimate the cut-off values, the Youden index (J) was calculated according to the following equation: J = sensitivity + (specificity-1). The cut-off values were those with maximum Youden index values.

In this study, rapid lactate clearance was defined as the lactate clearance above the threshold value or normalization of serum lactate level (<2.0 mmol/L) at 6 hours after a survival event Box 1. We used multivariable logistic regression to determine the effects of hypotension and the presence of rapid lactate clearance on the prediction of good outcomes during the early PCAC period, after adjusting for the selected covariates. Statistically significant variables with *p* < 0.1 were entered into a multivariable logistic regression model. We examined the incidence of liver cirrhosis because severe liver disease affects the clearance of serum lactate.

Values are presented as mean ± standard deviation (SD) for continuous variables and number (%) for categorical variables. Continuous variables were compared using an unpaired Student *t*-test or analysis of variance. When the normality assumption was violated (Kolmogorov-Smirnov normality test), continuous variables were compared using Mann–Whitney U nonparametric tests. Categorical variables were compared with chi-square or Fisher’s exact tests. All statistical analyses were carried out using IBM SPSS Statistics for Windows, version 20.0 (IBM Corp., Armonk, NY, USA). Two-tailed p-values of <0.05 were considered statistically significant.

## Results

### Comparisons of study population characteristics by outcome status

Data on a total of 1,312 patients with OHCA who received ACLS at the ED were included in the CPR registry during the study period. Of these, 603 patients had survival events, 335 of whom were enrolled in this study. We excluded 268 patients for age less than 18 years, cardiac arrest due to trauma, or lack of data for lactate levels at 0 and 6 hours after a survival event, resulting in 335 patients meeting the study’s inclusion criteria (Fig 1). Overall, 43.3% of the patients survived; 58 patients (17.3%) had a CPC score of 1 or 2 at discharge.

The baseline characteristics and CPR variables in each group are shown in Table 1. Compared with patients in the poor outcome group, those in the survivor group were significantly younger, more likely to have had an OHCA of suspected cardiac cause, and more frequently presented to the pre-hospital area with an initial shockable rhythm. The total CPR and ACLS durations Box 1 were significantly longer in the non-survivor group (*p* < 0.001, *p* < 0.001, respectively). The rates of TTM and CAG were higher in the survivor group than in the non-survivor group (*p* < 0.001, *p* < 0.001, respectively) (Table 1).

**Table 1:** Comparison of characteristics of the study population by outcome status.

	Survivors	Non-survivors	p-value
	(n = 145)	(n = 190)	
**Demographic factors**			
Age, years (IQR) *	62 (52.5–76)	70 (59–78)	0.004
Male:Female	90:55	113:77	0.653
Charlson Comorbidity Index score (IQR) *	1 (0–2)	1 (0–3)	0.327
**CPR-related factors**			
Arrest location (home), n (%)	65 (44.8)	95 (50.0)	0.378
Witnessed arrest, n (%)	107 (73.8)	132 (69.5)	0.396
Bystander CPR, n (%)	84 (57.9)	102 (53.7)	0.506
**Arrest cause**			0.008
**Presumed cardiac etiology, n (%)**	83 (57.2)	80 (42.1)	
**Non-cardiac etiology, n (%)**	62 (42.8)	110 (57.9)	
Respiratory origin	36	32	
CVA	4	13	
Hemorrhage	0	5	
Metabolic cause	8	25	
Sepsis/ terminal illness	5	15	
Intoxication	2	2	
Not recorded	7	18	
**Initial rhythm, n (%)**			<0.001
Shockable arrest rhythm, n (%)	52 (35.9)	23 (12.1)	
PEA	58 (40.0)	67 (35.3)	
Asystole	35 (24.1)	100 (52.6)	
**EMS response time (call to EMS arrival), min (IQR)** *	6 (5–8)	6 (5–7)	0.462
**Pre-hospital CPR duration, min (IQR)** *	10 (5–16)	16 (9–22)	<0.001
**In-hospital CPR duration, min (IQR)** *	8 (5.5–14.5) (n = 97)	12.5 (6–22) (n = 176)	0.043
**Arrest to survival event, min (IQR)** *	23 (13–36)	36.5 (27–49)	<0.001
**ACLS duration, min (IQR)** *	16 (8–26.5)	28.5 (20–38)	<0.001
**Post-resuscitation management**			
Targeted temperature management, n (%)	65 (44.8)	31 (16.3)	<0.001
Coronary angiography, n (%)	68 (46.9)	53 (27.9)	<0.001
**Mean arterial pressure (MAP) and lactate level**			
MAP (mmHg) at 0 hours	88 ± 28	68 ± 27	<0.001
MAP (mmHg) at 6 hours	87 ± 19	72 ± 24	<0.001
Vasoactive agent at 0 hours, n (%)	57 (39.3)	150 (78.9)	< 0.001
Vasoactive agent at 6 hours, n (%)	80 (55.2)	164 (86.3)	< 0.001
MAP < 65 mmHg at 0 hours, n (%)	34 (23.6)	106 (56.1)	< 0.001
MAP ≥ 65 mmHg at 0 hours without vasoactive agent, n (%)	74 (51.0)	24 (12.6)	< 0.001
MAP < 65 mmHg at 6 hours, n (%)	15 (10.4)	79 (41.8)	< 0.001
MAP ≥ 65 mmHg at 6 hours without vasoactive agent, n (%)	62 (42.8)	20 (10.5)	< 0.001
Serum lactate level (mmol/L) at 0 hours	9.2 ± 4.0	11.9 ± 5.1	<0.001
Serum lactate level (mmol/L) at 6 hours	5.0 ± 4.1	9.5 ± 5.7	<0.001
Rate of lactate clearance (%)	42 ± 36	18 ± 44	<0.001
**Outcome**			
24 hr survival, n (%)	145 (100)	95 (50)	
Hospital days, day (IQR) *	12 (3–21.5)	1 (0–4)	<0.001
CPC 1,2 at hospital discharge, n (%)	58 (40)	N/A	
CPC 1,2 at 3 months, n (%)	48 (33.1)	N/A	

Continuous variables are presented as mean ± SD or *median (interquartile ranges, IQR). Categorical variables are presented as number (%) of subjects

ACLS = advanced cardiac life support; CPR = cardiopulmonary resuscitation; MAP = mean arterial pressure; ROSC = return of spontaneous circulation.

The MAP in the survivor group was significantly higher than that in the non-survivor group at both 0 and 6 hours after a survival event (Table 1). The incidence of hypotension was also lower among survivors than non-survivors at both 0 and 6 hours.

Of the 1312 patients with OHCA, 1143 were adult OHCA patients with non-traumatic cause. Of these, 650 had ROSC and 493 did not. Among them, 452 of 650 patients with ROSC and 346 of 493 patients without ROSC had serum lactate level measurements during CPR. The mean serum lactate levels of patients during CPR with and without ROSC were 11.8 mmol/L (± 5.4) and 13.7 mmol/L (± 5.6), respectively (p < 0.001). In the study population, the serum lactate levels in the survivor group were significantly lower than those in the non-survivor group at both 0 and 6 hours (Table 1). The rate of lactate clearance among survivors was significantly higher than that among non-survivors (*p* < 0.001) at 6 hours after a survival event.

### Effect of lactate clearance rate on the outcomes of OHCA

**Fig 2:**
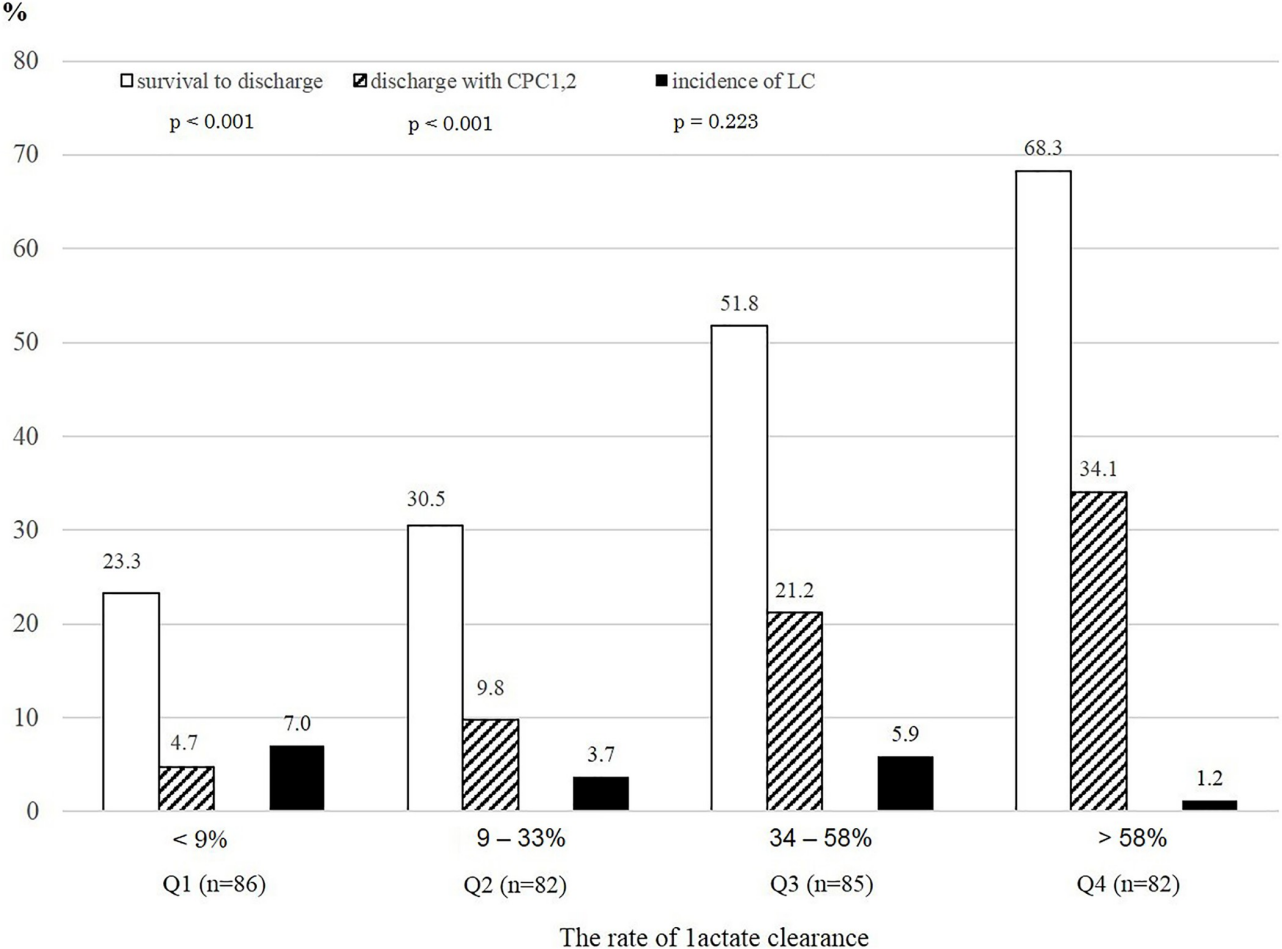
Effect of lactate clearance rate on survival and functional neurologic recovery rates. The lactate clearance rates were grouped by quartile (Q). Both the rate of survival to discharge and of CPC 1 or 2 at discharge increased with increasing lactate clearance rate (*p* < 0.001, respectively). There was no significant difference in the incidence of liver cirrhosis (LC) between the groups (*p* = 0.223). CPC, cerebral performance category.

Both the rate of survival to discharge and rate of CPC 1 or 2 at discharge increased with an increasing lactate clearance rate (*p* < 0.001, *p* < 0.001, respectively) (Fig 2). There was no significant difference in the incidence of liver cirrhosis according to the rate of lactate clearance (*p* = 0.223). Liver cirrhosis was judged based on the past medical history of patients with cardiac arrest. This study included 15 patients with liver cirrhosis. According to lactate clearance quartiles, six, three, five, and one patient had liver cirrhosis according to the classification (Fig 2).

**Fig 3:**
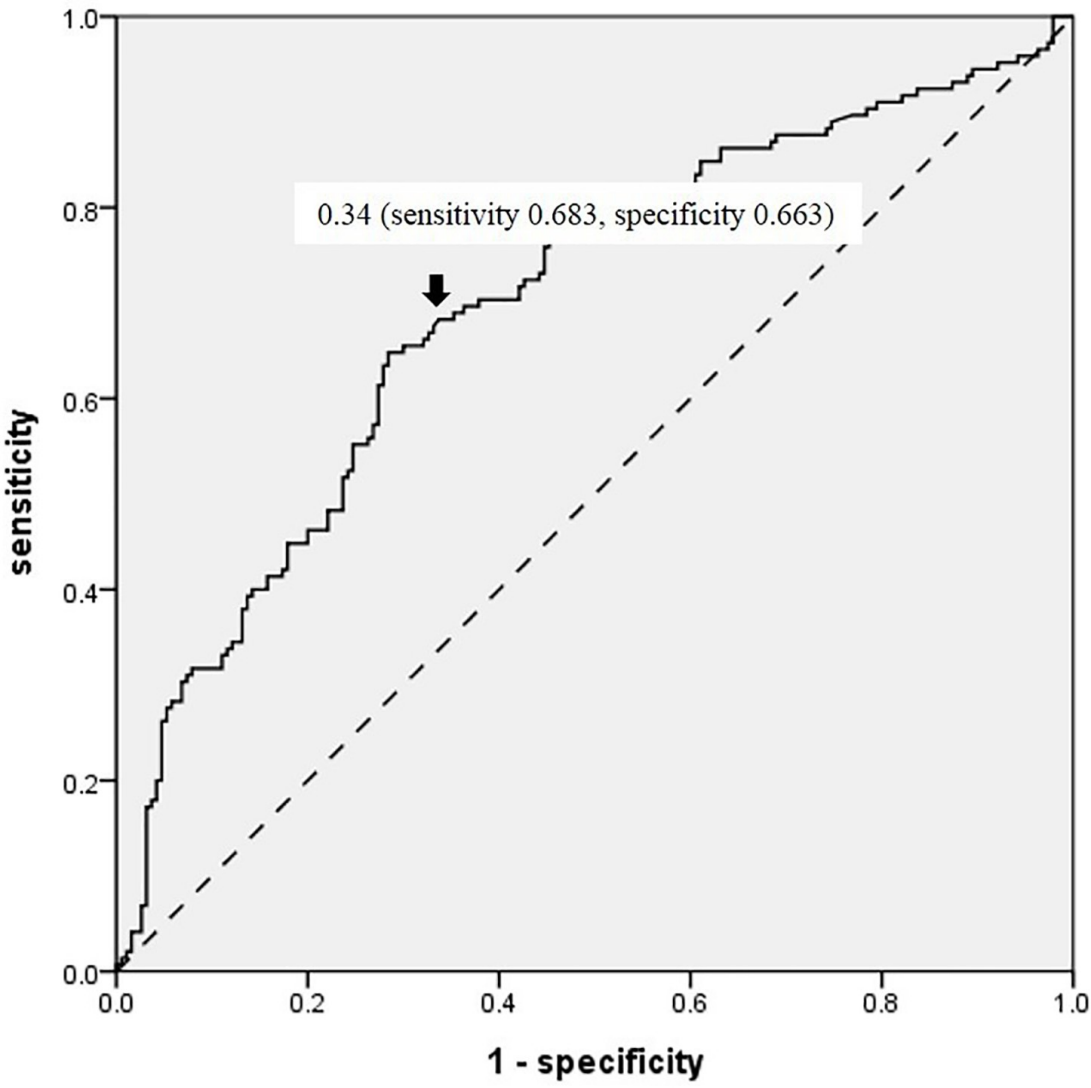
Receiver operator characteristic (ROC) curve of lactate clearance rate for prediction of survival to discharge. The threshold value of lactate clearance rate was determined at the point with maximum area under the ROC curve (AUC) 0.699 (0.642–0.756; p = 0.029).

**Fig 4:**
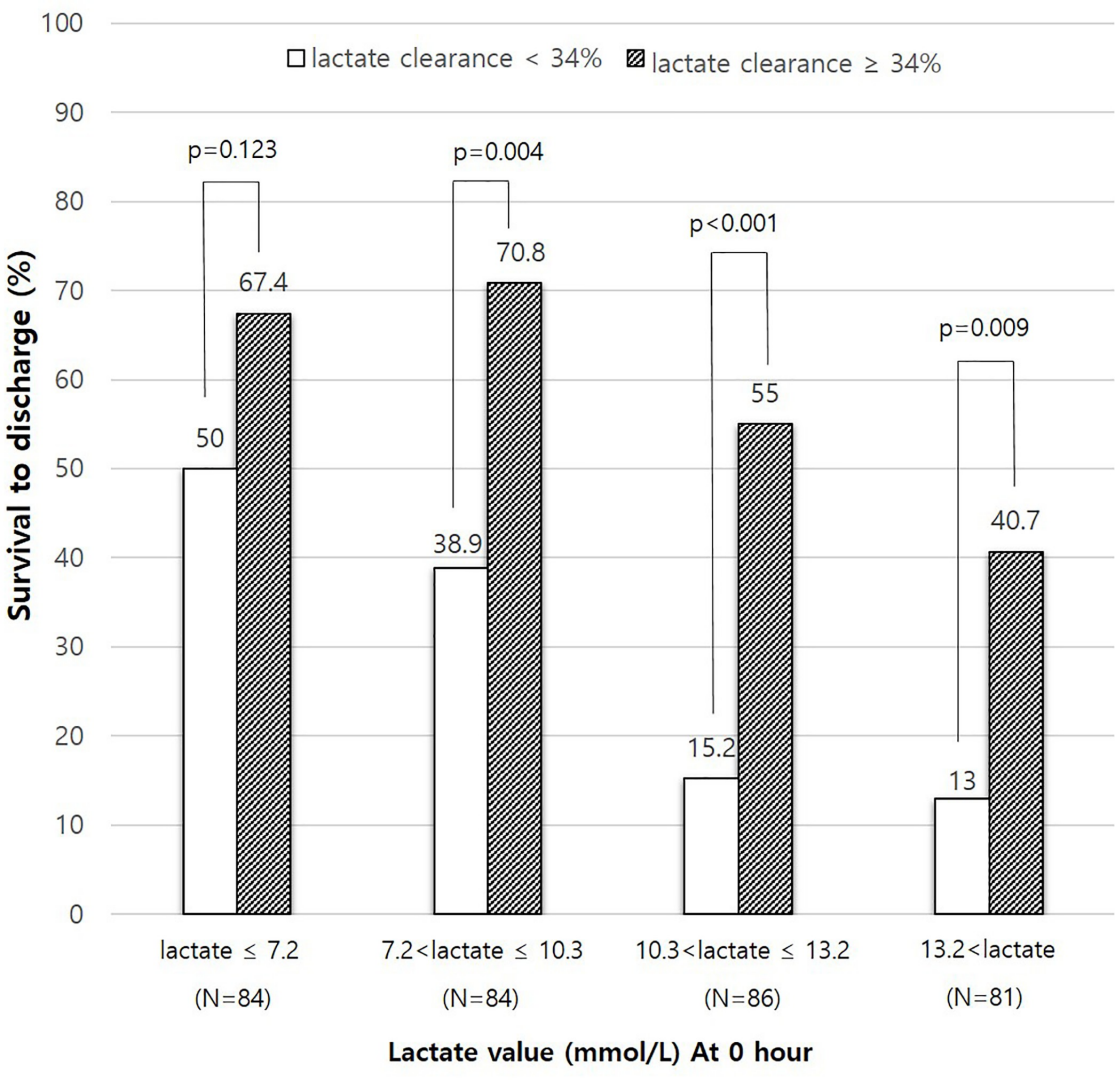
Effect of lactate clearance on survival to discharge according to quartiles of baseline lactate level.

The area under the ROC curve for the rate of lactate clearance was 0.699 (0.642–0.756, p = 0.029). The sensitivity and specificity for predicting survival to discharge were 68.3% and 66.3%, respectively, at a threshold value lactate clearance rate of 34% (Fig 3). A total of 169 (50.4%) patients had rapid lactate clearance at 6 hours after a survival event. The baseline lactate values (serum lactate level at 0 hours after survival) were divided into quartiles. Among patients with a higher baseline lactate level (above the second percentile group), the survival rates were significantly higher in the high lactate clearance group (≥34%) than in the low lactate clearance group (<34%) (Fig 4).

### Relationship between the presence of hypotension and outcomes of OHCA and lactate clearance rate

**Fig 5:**
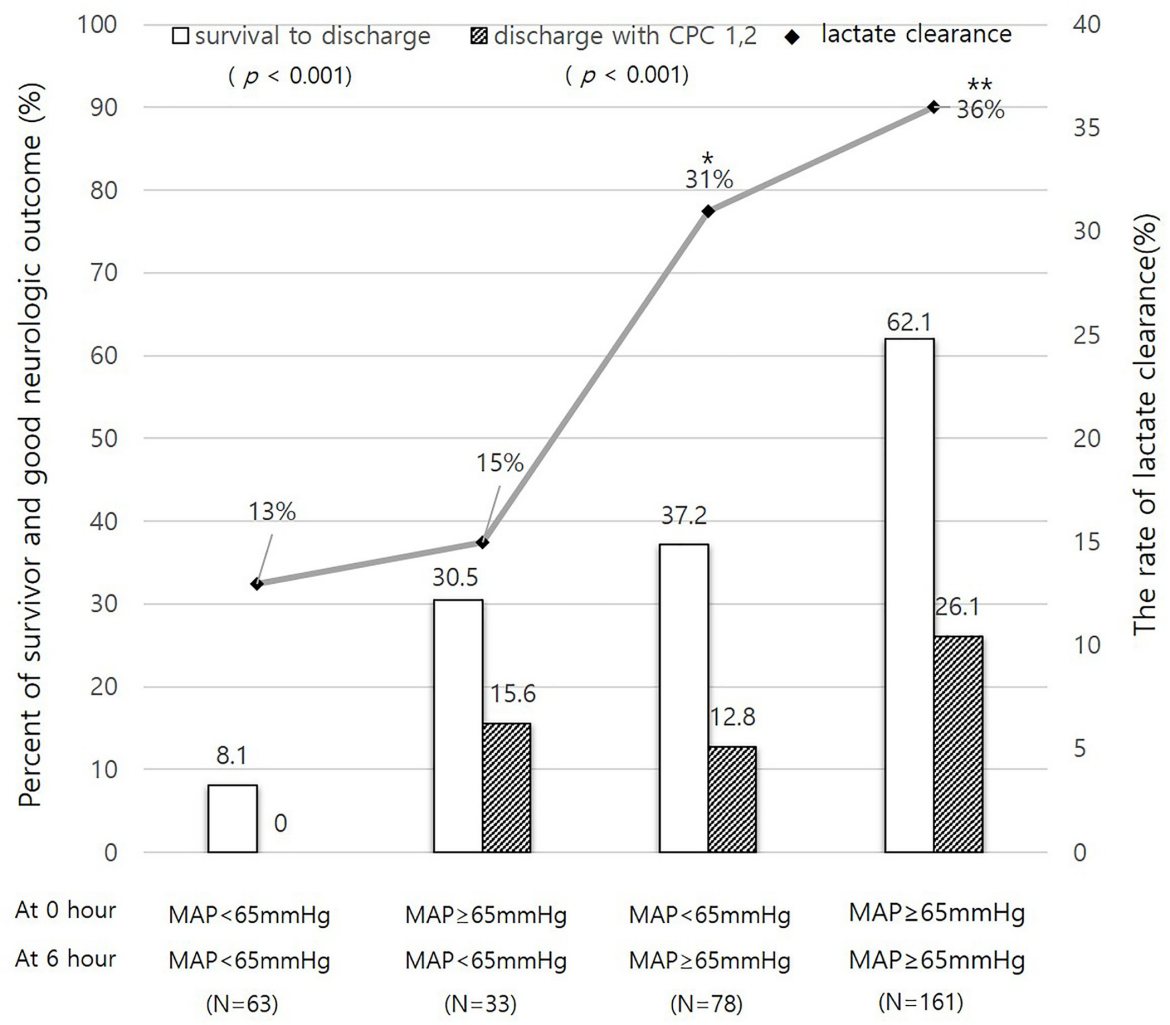
Changes in outcomes and lactate clearance according to the presence or absence of hypotension (mean arterial blood pressure [MAP] < 65 mmHg) at 0 and 6 hours after a survival event. The rates of survival and good neurologic outcome were significantly increased with the prevention or immediate correction of hypotension (*p* < 0.001 and *p* < 0.001), respectively). Lactate clearance rates in patients with early recovery from hypotension or with no hypotension events were higher than those in patients with persistent hypotension (*p = 0.064 and **p = 0.001, respectively). CPC, cerebral performance category.

Patients without hypotension at both 0 and 6 hours or who recovered from initial hypotension showed significantly higher rates of survival to discharge compared to in patients with persistent hypotension at both 0 and 6 hours (*p* < 0.001, respectively) (Fig 5). These patients also exhibited good neurologic outcome compared to patients with persistent hypotension (*p* < 0.001, *p* = 0.002, respectively) (Fig 5).

Patients without hypotension at both 0 and 6 hours or who recovered from hypotension at 6 hours tended to exhibit higher rates of lactate clearance than patients with persistent hypotension at both 0 and 6 hours (*p* < 0.001, *p* = 0.064, respectively) (Fig 5).

### Multivariable logistic regression analysis for the prediction of survival to discharge

After controlling for age, cause of arrest, initial rhythm, ACLS duration, PCAC management (TTM or CAG), change in MAP level (hypotension or no hypotension), and the presence of rapid lactate clearance at 6 hours, multivariable logistic regression revealed that initial shockable rhythm, short ACLS duration, application of TTM, no hypotension at both 0 and 6 hours, and presence of rapid lactate clearance at 6 hours were independent predictors of survival to discharge. The odds ratio (OR) of survival to discharge with no hypotension at both 0 and 6 hours was 8.018 (95% confidence interval (CI), 2.802–22.947; *p* < 0.001). The OR of survival to discharge for the presence of rapid lactate clearance at 6 hours was 2.193 (95% CI, 1.263–3.808; *p* = 0.005) (Table 2).

**Table 2:** Multivariable analysis for predicting good neurologic recovery in patients with OHCA.

	OR (95% confidence interval)	*p*-value
**Age**	0.988 (0.970–1.006)	0.186
**Resumed cardiac etiology**	1.390 (0.750–2.578)	0.296
**Initial rhythm**		
Asystole	Reference	
VF/VT	3.017 (1.347–6.756)	0.007
PEA	1.576 (0.853–2.912)	0.147
**ACLS duration**	0.965 (0.947–0.984)	<0.001
**Targeted temperature management**	2.283 (1.247–4.178)	0.007
**CAG**	1.030 (0.530–2.003)	0.93
**Change in MAP at 0 and 6 hours after**		
**a survival event**		
<65 to <65 mmHg	Reference	
≥65 to <65 mmHg	3.808 (1.041–13.934)	0.043
<65 to ≥65 mmHg	4.038 (1.330–12.258)	0.014
≥65 to ≥65 mmHg	8.018 (2.802–22.947)	<0.001
Rapid lactate clearance at 6 h* **(yes)**	2.193 (1.263–3.808)	0.005

*Rapid lactate clearance was defined as lactate clearance rate of ≥34% or normalization of serum lactate level (<2.0 mmol/L) at 6 hours after a survival event.

OHCA, out-of-hospital cardiac arrest; OR, odds ratio; VF, ventricular fibrillation; VT, ventricular tachycardia; PEA, pulseless electrical activity; ACLS, advanced cardiac life support; CAG, coronary angiography; MAP, mean arterial pressure.

### Comparisons of survival rates between rapid and non-rapid lactate clearance for each MAP status at 0 and 6 hours after a survival event

**Fig 6:**
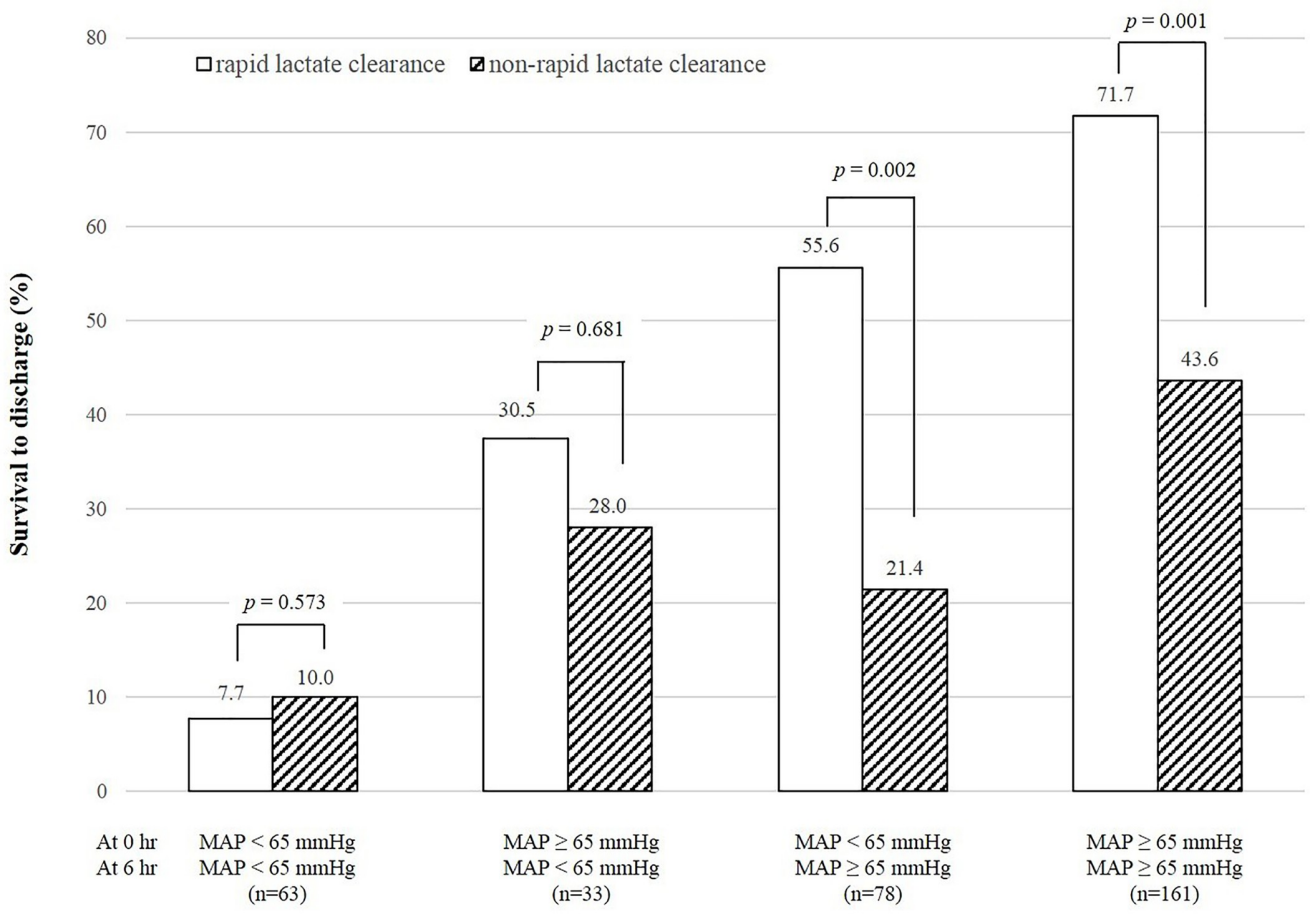
Comparisons of survival rates according to the presence or absence of rapid lactate clearance for each combination of hypotension (mean arterial blood pressure [MAP] < 65 mmHg) at 0 and 6 hours after a survival event. Among patients with early recovery from hypotension or with no hypotension events, the rate of survival to discharge differed significantly according to the presence of rapid lactate clearance. Rapid lactate clearance was defined as lactate clearance rate of ≥ 34% or normalization of serum lactate level (<2.0 mmol/L) at 6 hours after a survival event.

Among patients with early recovery from initial hypotension or with no hypotension events at both 0 and 6 hours, survival rates were significantly higher in the rapid lactate clearance group than in the non-rapid lactate clearance group (*p* = 0.002, *p* = 0.001, respectively) (Fig 6).

## Discussion

Adequate tissue perfusion and rapid reduction in lactate level during the PCAC period may be important indicators of hemodynamic optimization for the prediction of positive outcomes. We investigated the association between the presence of rapid lactate clearance and hypotension during the early post-cardiac arrest period as well as the survival rate. In this study, the presence of rapid lactate clearance during the first 6 hours of PCAC was significantly related to good outcomes in patients with OHCA. Immediate correction of hypotension or no hypotension at both 0 and 6 hours after a survival event was associated with not only good outcomes but also a higher rate of lactate clearance. Rapid lactate clearance at 6 hours after a survival event was associated with better outcomes in patients who recovered from initial hypotension or those who had no hypotension at both 0 and 6 hours than in patients without rapid lactate clearance.

Hemodynamic optimization, which includes providing organ perfusion and maintaining adequate blood pressure, is related to good outcomes in patients with OHCA. Serum lactate level reflects the status of tissue perfusion and is a prognostic marker for global tissue hypoxia in case of circulatory shock or arrest [2]. In our study, serum lactate levels were lower among patients in the survivor group than in the non-survivor group at both 0 and 6 hours after a survival event. Our results revealed a significantly higher lactate clearance rate at 6 hours after a survival event in the survival group. These findings are consistent with those of studies by Riveiro et al. and Hayashida et al. suggesting that lactate clearance rates in the first 6 hours after an arrest are associated with survival and good neurologic outcomes [3, 12].

Our study showed that survivors had higher MAP values at both 0 hours and 6 hours after a survival event than non-survivors. Beylin et al. demonstrated that survivors had higher MAP values (mean MAP, 94–96 mmHg) than non-survivors in the early PCAC period (1 and 6 hours after ROSC) with or without the use of vasoactive agents [13]. Kilgannon et al. suggested that an average MAP level of >70 mmHg in the first 6 hours following resuscitation was associated with good neurologic outcomes [9]. Our study showed higher mortality in patients with persistent hypotension at both 0 and 6 hours. Although the optimal MAP during the PCAC period has not been well established in OHCA, international guidelines recommend prevention and immediate correction of any incidence of hypotension (systolic blood pressure < 90 mmHg or MAP < 65 mmHg) [10]. Similarly, Young et al. reported that hypotension at admission was related to poor neurologic outcomes; however, the authors reported that the achievement of a higher MAP during therapeutic hypothermia was not associated with good neurologic outcomes at discharge [14].

The true optimal blood pressure would be a level that allows for optimal organ and brain perfusion [10]. The initial lactate levels of patients after an arrest are usually elevated due to global ischemia during the arrest; however, optimal organ perfusion under optimal blood pressure may decrease the production of serum lactate and rapidly reduce the serum lactate level. Masyuk et al. reported that the change in lactate levels for 24 hours after admission was associated with survival in critically ill patients [15]. Changes in lactate level over time reflect changes in production and elimination [16]. In this study, the rate of lactate clearance was not associated with the incidence of liver cirrhosis, which can decrease lactate clearance. Therefore, slow lactate clearance during the early PCAC period may reflect inadequate tissue perfusion and impaired microcirculation, which may result in a lower survival rate when systemic macro-hemodynamic parameters are within the normal ranges [17]. We must consider preventing and immediately correcting hypotension and rapid lactate clearance as indicators of hemodynamic optimization.

The results of our study are similar to the previously reported hemodynamic goals. However, we have proposed a cut-off for lactate clearance, which currently lacks a clear standard, and our results suggest that patients with high lactate clearance may have a better prognosis, even if the blood pressure is maintained in the early PCAC period. However, additional research is needed on how to increase lactate clearance.

This study has several limitations. First, this retrospective, descriptive analysis was based on data from a single center. The small sample size limited the generalization of the results. Second, selection bias was possible due to patients excluded for missing lactate values. This study excluded 199 patients with missing lactate values at 0 or 6 hours. However, there was no statistical difference in the outcome of survival discharge and good neurological prognosis in the groups included and not included in the study (S1 Table). Third, TTM was not applied to all patients because it takes time to implement and apply TTM and there have been changes in the ACLS guidelines. Before 2010, TTM was not used frequently. After the presentation of the 2010 AHA guideline, TTM was more frequently applied until 2015 (S2 Table). TTM has been associated with ischemic and reperfusion injury and may affect lactate clearance. Therefore, one disadvantage of the present study is that it did not consider the implementation of TTM. Fourth, the study duration was more than 11 years; thus, there were changes in treatment according to guideline changes. The patient characteristics were compared based on the 2010 and 2015 guidelines. TTM showed the highest rate from 2010 to 2015. Over time, the frequency of witnessed CPR and CAG administration increased. However, there was no statistically significant difference in the outcome according to the period (S2 Table). Lastly, the effect of vasoactive agents on variables was not considered. However, in clinical settings, hemodynamic parameters such as MAP are usually compensated for with the use of vasoactive agents; we sought to determine the impact of rapid lactate clearance and trends associated with hypotension rather than the absolute values. Laurikkala et al. suggested that the interaction between hypotension and use of vasoactive agents was not associated with neurologic outcomes [18]. Further multicenter studies are required that consider factors such as the use of vasoactive agents and the presence of precipitating diseases.

## Conclusions

During the first 6 hours after a survival event, a lactate clearance rate of 34% or more and normalization of the serum lactate level were strongly associated with a good outcome in patients with OHCA. We found that preventing hypotension was an independent predictor of better neurologic outcomes. In particular, preventing hypotension with rapid lactate clearance in the early PCAC period showed better survival rates in patients with OHCA. Hemodynamic optimization that includes not only the prevention and immediate correction of hypotension but also rapid lactate clearance should be considered to improve outcomes in patients with OHCA.

## Supporting information

S1 File STROBE checklist. (DOCX)

S1 Table Comparisons of the characteristics and outcomes of included and excluded patients. (DOCX)

S2 Table Comparisons of characteristics and outcomes according to guideline changes. (DOCX)
