# Supplementary material for: Impact of rapid lactate clearance as an indicator of hemodynamic optimization on outcome in out-of-hospital cardiac arrest: A retrospective analysis
Source: PLoS One. 2019 Apr 1;14(4):e0214547. doi: 10.1371/journal.pone.0214547 (PMC6443161; doi:10.1371/journal.pone.0214547)
Supplement: S1 File — (DOCX) [file pone.0214547.s001.docx]

**STROBE Checklist, retrospective cross sectional study**

|  | Item No | Recommendation | Section paragraph(s) |
| --- | --- | --- | --- |
| **Title and abstract** | 1 | (*a*) Indicate the study’s design with a commonly used term in the title or the abstract. | page 1 in the title section |
|  |  | (b) Provide in the abstract an informative and balanced summary of what was done and what was found | Page 3 in the abstract section |
| Introduction | | |  |
| Background/rationale | 2 | Explain the scientific background and rationale for the investigation being reported | Page 4 in the introduction section |
| Objectives | 3 | State specific objectives, including any prespecified hypotheses | Page 4, in the introoduction section |
| Methods | | |  |
| Study design | 4 | Present key elements of study design early in the paper | Page 4-5, in the method section |
| Setting | 5 | Describe the setting, locations, and relevant dates, including periods of recruitment, exposure, follow-up, and data collection | Page 5, in the method section |
| Participants | 6 | (*a*) Give the eligibility criteria, and the sources and methods of selection of participants. Describe methods of follow-up | Page 5-6, in the method section |
|  |  | (*b*) For matched studies, give matching criteria and number of exposed and unexposed | NA |
| Variables | 7 | Clearly define all outcomes, exposures, predictors, potential confounders, and effect modifiers. Give diagnostic criteria, if applicable | Page 5-6, in the method section |
| Data sources/ measurement | 8* | For each variable of interest, give sources of data and details of methods of assessment (measurement). Describe comparability of assessment methods if there is more than one group | Page 5, in the method section |
| Bias | 9 | Describe any efforts to address potential sources of bias | Page 5, in the method section |
| Study size | 10 | Explain how the study size was arrived at | NA(retrospective observational study) |
| Quantitative variables | 11 | Explain how quantitative variables were handled in the analyses. If applicable, describe which groupings were chosen and why | Page 6, in the method section |
| Statistical methods | 12 | (*a*) Describe all statistical methods, including those used to control for confounding | Page 6, in the method section |
|  |  | (*b*) Describe any methods used to examine subgroups and interactions | Page 6, in the method section |
|  |  | (*c*) Explain how missing data were addressed | Page 7, in the result section |
|  |  | (*d*) If applicable, explain how loss to follow-up was addressed | NA |
|  |  | (*e*) Describe any sensitivity analyses | NA |
| Results | | |  |
| Participants | 13* | (a) Report numbers of individuals at each stage of study—eg numbers potentially eligible, examined for eligibility, confirmed eligible, included in the study, completing follow-up, and analysed | Page 7, in the result section Figure 1 |
|  |  | (b) Give reasons for non-participation at each stage | Page 7, in the result section Figure 1 |
|  |  | (c) Consider use of a flow diagram | Figure 1 |
| Descriptive data | 14* | (a) Give characteristics of study participants (eg demographic, clinical, social) and information on exposures and potential confounders | Page 7-8, in the result section Table 1 |
|  |  | (b) Indicate number of participants with missing data for each variable of interest | Page 7, in the result section Figure 1 |
|  |  | (c) Summarise follow-up time (eg, average and total amount) | NA |
| Outcome data | 15* | Report numbers of outcome events or summary measures over time | Figure 1 |
| Main results | 16 | (*a*) Give unadjusted estimates and, if applicable, confounder-adjusted estimates and their precision (eg, 95% confidence interval). Make clear which confounders were adjusted for and why they were included | Page 8-9, in the result section |
|  |  | (*b*) Report category boundaries when continuous variables were categorized | Page 6, in the method section |
|  |  | (*c*) If relevant, consider translating estimates of relative risk into absolute risk for a meaningful time period | Page 8-9, in the result section Table 2 |
| Other analyses | 17 | Report other analyses done—eg analyses of subgroups and interactions, and sensitivity analyses | Page 9-10, in the result section Figure 3,4 |
| Discussion | | |  |
| Key results | 18 | Summarise key results with reference to study objectives | Page 11, in the discussion section |
| Limitations | 19 | Discuss limitations of the study, taking into account sources of potential bias or imprecision. Discuss both direction and magnitude of any potential bias | Page 12, in the discussion section |
| Interpretation | 20 | Give a cautious overall interpretation of results considering objectives, limitations, multiplicity of analyses, results from similar studies, and other relevant evidence | Page 12-13, in the discussion section |
| Generalisability | 21 | Discuss the generalisability (external validity) of the study results | NA |
| Other information | | |  |
| Funding | 22 | Give the source of funding and the role of the funders for the present study and, if applicable, for the original study on which the present article is based.  We have included this information in our financial disclosure: | NA |
