# Supplementary material for: Impact of rapid lactate clearance as an indicator of hemodynamic optimization on outcome in out-of-hospital cardiac arrest: A retrospective analysis
Source: PLoS One. 2019 Apr 1;14(4):e0214547. doi: 10.1371/journal.pone.0214547 (PMC6443161; doi:10.1371/journal.pone.0214547)
Supplement: S1 Table — (DOCX) [file pone.0214547.s002.docx]

**S1 Table. Comparisons of the characteristics and outcomes of included and excluded patients.**

|  | **Included patients** | **Excluded patients**  **d/t no lactate value at 0 or 6 h** | ***p*-value** |
| --- | --- | --- | --- |
|  | (n = 335) | (n = 199) |  |
| **Demographic factors** |  |  |  |
| Age (years) | 65.1 ± 15.6 | 65.3 ± 15.3 | 0.885 |
| Male:Female | 203:132 | 130:69 | 0.310 |
| Charlson Comorbidity Index score | 1 (0-2) | 1 (0-3) | 0.314 |
| **CPR-related factors** |  |  |  |
| Arrest location (home), n (%) | 160 (47.8) | 114 (57.3) | 0.039 |
| Witnessed arrest, n (%) | 239 (71.3) | 162 (81.4) | 0.010 |
| Bystander CPR, n (%) | 186 (55.5) | 113 (56.8) | 0.788 |
| Presumed cardiac etiology, n (%) | 163 (48.7) | 129 (64.8) | 0.000 |
| Shockable arrest rhythm, n (%) | 70 (21.0) | 60 (30.3) | 0.022 |
| Arrest to survival event (min) | 31 (19-44) | 31 (15-43) | 0.314 |
| ACLS duration (min) | 24 (14-35) | 23 (10-30) | 0.197 |
| **Post-resuscitation management** |  |  |  |
| Targeted temperature management, n (%) | 96 (28.7) | 33 (16.6) | 0.002 |
| Coronary angiography, n (%) | 121 (36.1) | 80 (40.7) | 0.311 |
| **Mean arterial pressure (MAP) and lactate** |  |  |  |
| MAP (mmHg) at 0 hours | 76 ± 29 | 79 ± 31 | 0.409 |
| MAP (mmHg) at 6 hours | 78 ± 23 | 81 ± 24 | 0.156 |
| **Outcome** |  |  |  |
| Survival to hospital discharge (%) | 43.3 | 43.7 | 0.928 |
| CPC 1,2 at hospital discharge (%) | 17.3 | 23.1 | 0.114 |

Continuous variables are presented as mean ± SD or median (interquartile ranges). Categorical variables are presented as number (%) of subjects

ACLS= advanced cardiac life support; CPR= cardiopulmonary resuscitation; MAP=mean arterial pressure; ROSC= return of spontaneous circulation.
