# Supplementary material for: Impact of rapid lactate clearance as an indicator of hemodynamic optimization on outcome in out-of-hospital cardiac arrest: A retrospective analysis
Source: PLoS One. 2019 Apr 1;14(4):e0214547. doi: 10.1371/journal.pone.0214547 (PMC6443161; doi:10.1371/journal.pone.0214547)
Supplement: S2 Table — (DOCX) [file pone.0214547.s003.docx]

**S2 Table. Comparisons of characteristics and outcomes according to guideline changes.**

| **Study population**  **N=1143** | **2006-2010** | **2011-2015** | **2016-2017** | **p-value** |
| --- | --- | --- | --- | --- |
|  | (n = 35) | (n = 216) | (n = 84) |  |
| **Demographics factors** |  |  |  |  |
| Age (years) | 65.3 15.2 | 64.4 15.6 | 66.8 15.6 | 0.475 |
| Male:Female | 22:13 | 136:80 | 45:39 | 0.314 |
| Charlson Comorbidity Index score | 1(0-2) | 1(0-3) | 1(0-3) | 0.275 |
| **CPR-related factors** |  |  |  |  |
| Arrest location (home), n (%) | 15 (42.9) | 102 (47.2) | 43 (51.2) | 0.087 |
| Witnessed arrest, n (%) | 25 (71.4) | 154 (71.3) | 60 (71.4) | 1.000 |
| Bystander CPR, n (%) | 5 (14.3) | 127 (58.8) | 54 (64.3) | <0.001 |
| Presumed cardiac etiology, n (%) | 16 (45.7) | 108 (50.0) | 39 (46.4) | 0.801 |
| Shockable arrest rhythm, n (%) | 6 (17.1) | 53 (24.5) | 16 (19.0) | 0.710 |
| Arrest to survival event (min) | 34 (26-52) | 31 (18-43) | 32 (17-47) | 0.392 |
| ACLS duration | 25 (17-37) | 23 (14-34) | 26 (10-36) | 0.359 |
| **Post-resuscitation management** |  |  |  |  |
| Targeted temperature management, n (%) | 2 (5.7) | 75 (34.7) | 19 (22.6) | 0.001 |
| Coronary angiography, n (%) | 6 (17.1) | 81 (37.5) | 34 (40.5) | 0.042 |
| **Outcomes** |  |  |  |  |
| 24 hr survival, n (%) | 20 (57.1) | 143 (66.2) | 49 (58.3) | 0.325 |
| Survival to hospital discharge (%) | 14 (40.0) | 101 (46.8) | 30 (35.7) | 0.204 |
| CPC 1,2 at hospital discharge (%) | 4 (11.4) | 37 (17.1) | 17 (20.2) | 0.508 |

Continuous variables are presented as mean ± SD or median (interquartile ranges). Categorical variables are presented as the number (%) of subjects

ACLS= advanced cardiac life support; CPR= cardiopulmonary resuscitation; MAP=mean arterial pressure; ROSC= return of spontaneous circulation.
